# Expression of the MT1 Melatonin Receptor in Ovarian Cancer Cells

**DOI:** 10.3390/ijms151223074

**Published:** 2014-12-12

**Authors:** Karolina Jablonska, Bartosz Pula, Agata Zemla, Christopher Kobierzycki, Witold Kedzia, Ewa Nowak-Markwitz, Marek Spaczynski, Maciej Zabel, Marzenna Podhorska-Okolow, Piotr Dziegiel

**Affiliations:** 1 Department of Histology and Embryology, Wrocław Medical University, Wrocław 50-368, Poland; E-Mails: karolina.jablonska@umed.wroc.pl (K.J.); bartosz.pula@gmail.com (B.P.); agatazemla@op.pl (A.Z.); ch.kobierzycki@gmail.com (C.K.); maciej.zabel@umed.wroc.pl (M.Z.); marzenna.podhorska-okolow@umed.wroc.pl (M.P.-O.); 2 Department of Physiotherapy, University School of Physical Education, Wrocław 51-612, Poland; 3 Department of Gynecology, University of Medical Sciences, Poznań 60-535, Poland; E-Mail: witold.kedzia@poczta.fm; 4 Department of Gynecological Oncology, University of Medical Sciences, Poznań 60-535, Poland; E-Mails: ewamarkwitz@poczta.fm (E.N.-M.); m.spaczynski@op.pl (M.S.); 5 Department of Histology and Embryology, University of Medical Sciences, Poznań 60-781, Poland

**Keywords:** melatonin, melatonin receptor, ovarian cancer

## Abstract

Ovarian cancer (OC) is the leading cause of death among women with genital tract disorders. Melatonin exhibits oncostatic properties which it may effect through binding to its membrane receptor, MT1. The aim of this study was to determine the expression of MT1 in OC cells and to correlate this with clinical and pathological data. Immunohistochemistry was performed on 84 cases of OC. Normal ovarian epithelial (IOSE 364) and OC (SK-OV-3, OVCAR-3) cell lines were used to examine the MT1 expression at protein level using the western blot and immunofluorescence technique. The expression of MT1 was observed as cytoplasmic-membrane (MT1_CM_) and membrane (MT1_M_) reactions. A positive correlation between MT1_CM_ and MT1_M_ was found in all the studied cases. There were no significant differences between the expression of MT1_CM_, MT1_M_, and histological type, staging, grading, presence of residual disease, or overall survival time. Immunofluorescence showed both MT1_M_ and MT1_CM_ expression in all the tested cell lines. Western blot illustrated the highest protein level of MT1 in IOSE 364 and the lowest in the OVCAR-3. The results indicate the limited prognostic significance of MT1 in OC cells.

## 1. Introduction

Ovarian cancer (OC) is the most common malignancy of the female genital organs [1] and is the cause of death of more than 140,000 women per year worldwide [1,2]. More than 70 percent of OC cases are diagnosed at an advanced stage (III or IV) of the disease [3]. The most common risk factors for increased incidence of OC are number of pregnancies, duration of menopause, oral hormonal contraception, genetic factors, and age [4]. There are five basic histological types of OC: serous, mucinous, endometrioid, clear-cell, and undifferentiated [5]. These types differ in histogenesis, morphology, and clinical image [1,5,6].

Melatonin (Mel) is a hormone secreted in mammals, mainly by the pineal gland, in response to light information [7,8]. It is a chronobiotic molecule that regulates the circadian rhythm of the body [7,8]. Roles for Mel in insomnia, diabetes, Alzheimer’s disease, immune diseases, cardiac diseases, and neoplasms have also been reported [9]. There is an evidence that Mel has an antiproliferative effect on various types of neoplasms, such as breast [8,10], ovarian [11,12], prostate [13], and cervical cancers [14], as well on melanomas [15,16,17], lymphomas [18], and neuroblastomas [19]. The literature points to the oncostatic potential of Mel especially in estrogen-receptor positive (ER+) cells [8,20,21]. The mechanisms responsible for this inhibitory effect are still unclear. Mel has been shown to exert it effect on cells by affecting several pathways, ranging from antioxidant properties, binding to calmodulin and acting through its receptors [8,11,16,17,22]. Melatonin receptors have been classified on the basis of kinetic and pharmacological properties into two classes—ML1 and ML2. ML1 is a group of high-affinity membrane receptors which can be divided into three subtypes: MT1 (Mel1a), MT2 (Mel1b) and Mel1c. MT1 and MT2 are expressed in mammals whereas Mel1c was detected only in birds and *Xenopus laevis* [23]. ML2 group (now called MT3) is represented by one type of a low-affinity receptor which has been described in hamster as the human homologue of the cytoplasmic enzyme, quinone reductase 2 [24]. There is also hypothesis that Mel may act with nuclear orphan receptors from the retinoid-related orphan receptor α/retinoid Z receptor α (RORα/RZR) family but whether this hormone interacts directly with nuclear receptor remain still controversial. Recent reports suggest that RORα is a receptor for sterols and vitamin D hydroxyl derivatives not for melatonin [25]. The evidence that human breast cancer cells express MT1 but not MT2 create a hypothesis that the MT1 is responsible for melatonin’s oncostatic effect [26]. Additionally, the transfection of MT1 to MCF-7 cells (ER positive), MDA-MB-231 cells (ER negative), Chinese hamster ovary (CHO) cells lines and BE(2)C nuroblastoma human cells significantly increased efficiency of melatonin’s action. In context of the antiproliferative activity of Mel we decided to verify the location and intensity of the MT1 expression in ovarian cancer cells [27,28]. Mel receptors belong to the G-coupled transmembrane proteins [7,8]. Through activation of the MT1, Mel decreases cAMP synthesis via adenylyl cyclase inhibition, as well by activity depletion of the protein kinase C (PKC), protein kinase A (PKA), and mitogen-activated protein kinases (MAPK) [7,8,22]. This relationship has a negative influence on the phosphorylation of transcription factor CREB (cAMP response element-binding) and on the expression of the genes involved in proliferation, angiogenesis, and migration processes [22].

It was found in the 1960s that exogenous Mel decreases the weight of rats’ ovaries [29]. Mel and its metabolites exert a direct effect on the human reproductive system by influencing the function of the ovaries [30]. The reduction of Mel levels by pinealectomy (pineal gland excision) has been shown to impact ovarian morphology [31]. High concentrations of Mel have been also identified in human preovulatory follicular fluid [32]. Mel receptors have been localized in human granulosa cells [33], in rat antral follicles, and in the corpus luteum [31]. Furthermore, higher binding of [^0125^I]-iodomelatonin has been observed in proestrus ovarian tissues than in metestrus ovarian tissues, suggesting the association of Mel with estrogens [31].

Most studies have focused on the role of Mel in the regulation of reproduction processes and ovary function; however, little attention has been paid so far to the role of this hormone and its receptors in OC. Previous *in vitro* and *in vivo* studies of the expression of MT1 in OC cells have been inconclusive [12,34,35,36]. This prompted us to determine the expression of MT1 in this malignancy with special emphasis on its impact on patients’ clinical and pathological characteristics.

## 2. Results and Discussion

### 2.1. Results

**Figure 1:**
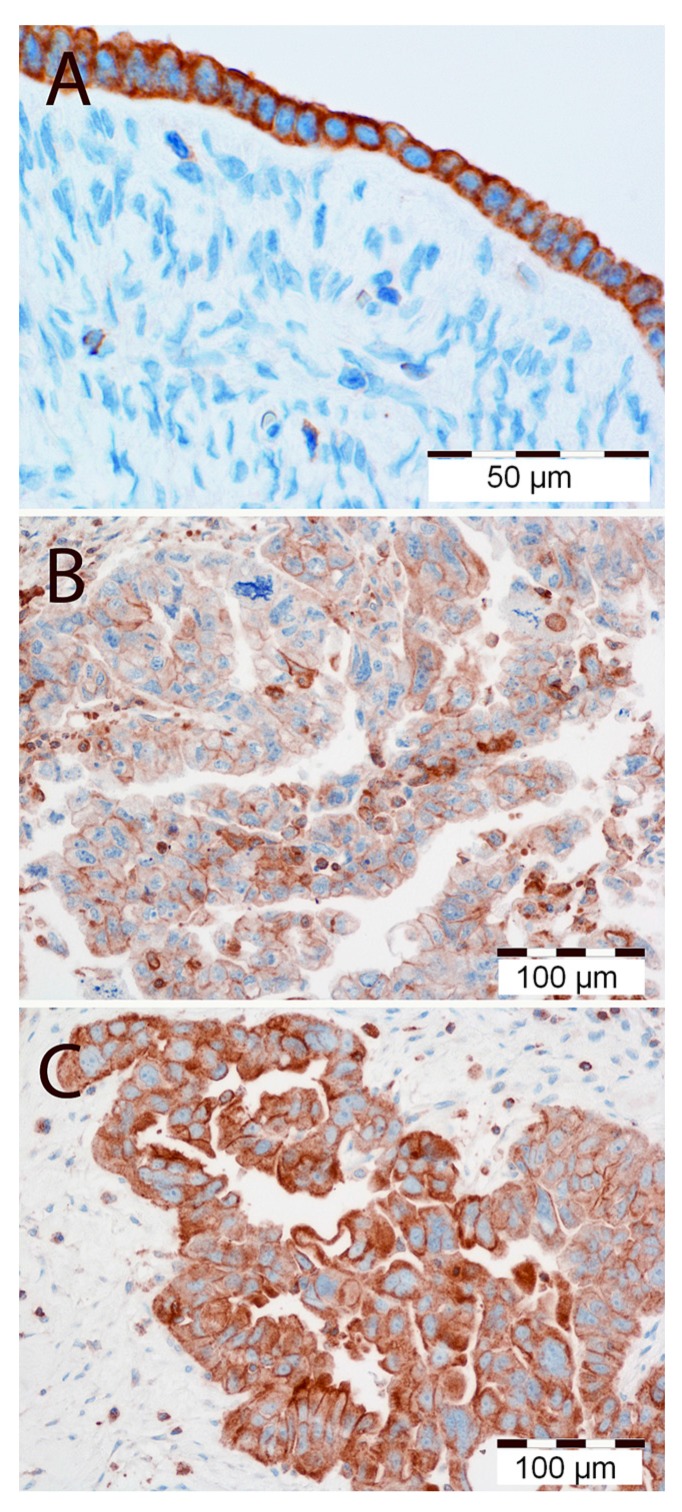
(**A**) High immunohistochemical cytoplasmic/membranous expression of MT1 in normal human ovarian surface epithelium. Magnification ×300. Low (**B**) and high (**C**) immunohistochemical cytoplasmic/membranous expression of MT1 in ovarian cancer cells. Magnification ×200. Scale bar = 50 μm (**A**), 100 μm (**B**,**C**).

MT1 expression was disclosed as cytoplasmic/membrane (MT1_CM_) or membrane (MT1_M_) fraction in all the studied samples. In the predominant number of cases—taking the whole study group (WG) and the serous type (SG) separately—strong MT1_CM_ expression (48 out of 84 and 35 out of 65, respectively) and MT1_M_ expression (52 out of 84 and 38 out of 65, respectively) was observed (Figure 1B,C). High expression of MT1 was also observed in the cells of normal human ovarian surface epithelium (Figure 1A). The mean values of the analyzed fractions, according to the evaluation scales presented here, were 5.42 ± 3.98 points for MT1_CM_ and 1.49 ± 1.54 points for MT1_M_. A strong positive correlation was noted between the MT1_CM_ and MT1_M_ expression intensity in the WG (*r* = 0.85, *p* < 0.001) and the SG (*r* = 0.83, *p* < 0.001). In the SG alone, close to statistical significance a positive correlation of MT1_M_ and Ki-67 (*r* = 0.218, *p* = 0.081) was observed. The expression of MT1_CM_ (*p* = 0.015) and MT1_M_ (*p* = 0.002) was significantly stronger in patients older than 50 years, while a significantly decreased expression of MT1_M_ (*p* = 0.0141) and Ki-67 (*p* = 0.0069) was found in regard to lymph node status in pN-negative cases. Moreover, significant differences in Ki-67 were found between G1 and G3 in the WG (*p* = 0.0237) and SG (*p* = 0.0278). No other statistically significant associations were shown between the expression of all tested markers in relation to malignancy grade, clinical advancement stage, tumor type, and presence of residual disease. Univariate Cox proportional hazards analysis disclosed a lack of impact of MT1 on the overall survival (OS) of patients from WG and SG. The expression levels of MT1_CM_ and MT1_M_ were not associated with patient survival time (Table 1). Advanced clinical stage, high expression of Ki-67 antigen, and the presence of residual disease following cytoreduction were all associated with poor OS in WG and SG (Table 1).

**Table 1:** Univariate Cox proportional hazard analysis in 84 patients with ovarian cancer.

Clinical and Pathological Parameters	OS					
	All			Patients with Serous Tumors		
	HR	95% CI	*p*-Value	HR	95% CI	*p*-Value
MT1_CM_ (“low” *vs.* “high”)	0.9384	0.5648–1.559	0.806	0.8742	0.5080–1.504	0.6274
MT1_M_ (“low” *vs.* “high”)	1.326	0.7778–2.262	0.2997	1.216	0.693–2.132	0.4959
Ki-67 (“low” *vs.* “high”)	0.5927	0.3525–0.9964	**0.0484**	0.4273	0.2354–0.7758	**0.0052**
Stage (I–II *vs.* III–IV)	1.799	1.039–3.114	**0.0360**	1.799	0.9547–3.391	0.0693
Tumor grade (G1–G2 *vs.* G3)	1.019	0.6147–1.689	0.9425	1.165	0.6728–2.018	0.5852
Age (≤50 *vs.* >50)	1.115	0.6674–1.863	0.6777	1.335	0.7575–2.352	0.9987
Cytoreduction (“optimal” *vs.* “nonoptimal”)	3.258	1.926–5.512	**<0.0001**	2.662	1.482–4.783	**0.0011**

Significant *p*-values are given in bold print. HR: hazard ratio; CI: confidence interval; OS: overall survival.

**Figure 2:**
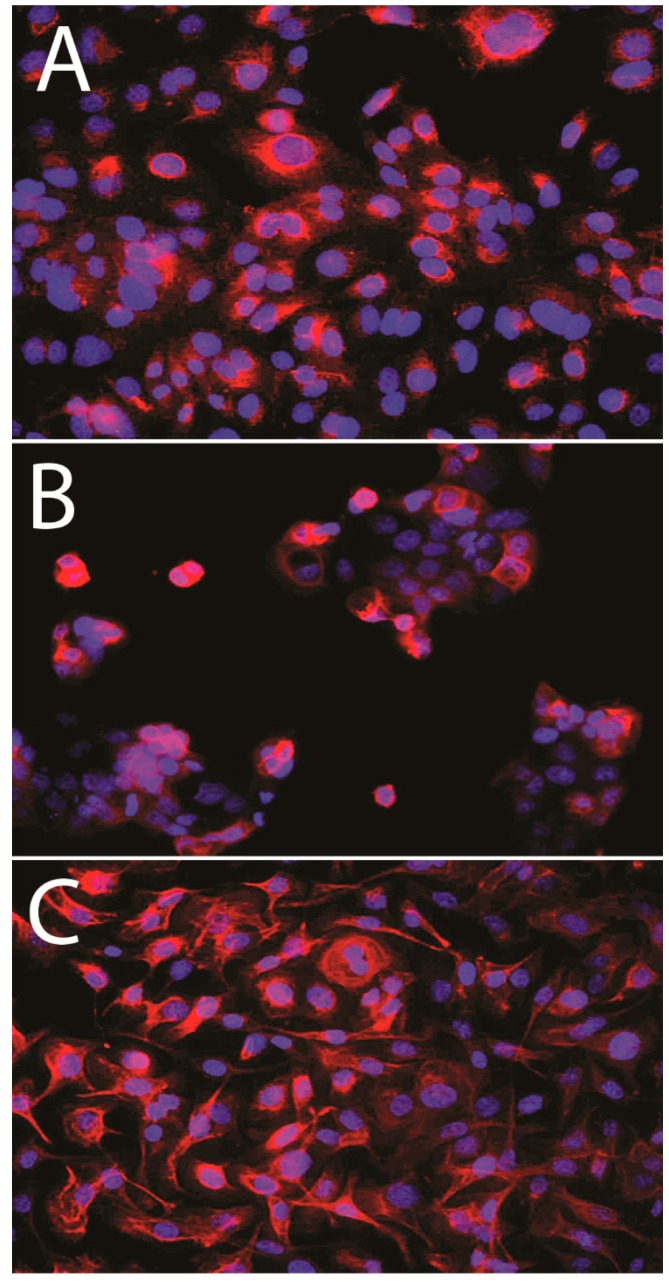
MT1 expression in membrane and cytoplasm of IOSE 364 (**A**); SK-OV-3 (**B**); OVCAR-3 (**C**) cell lines.

**Figure 3:**
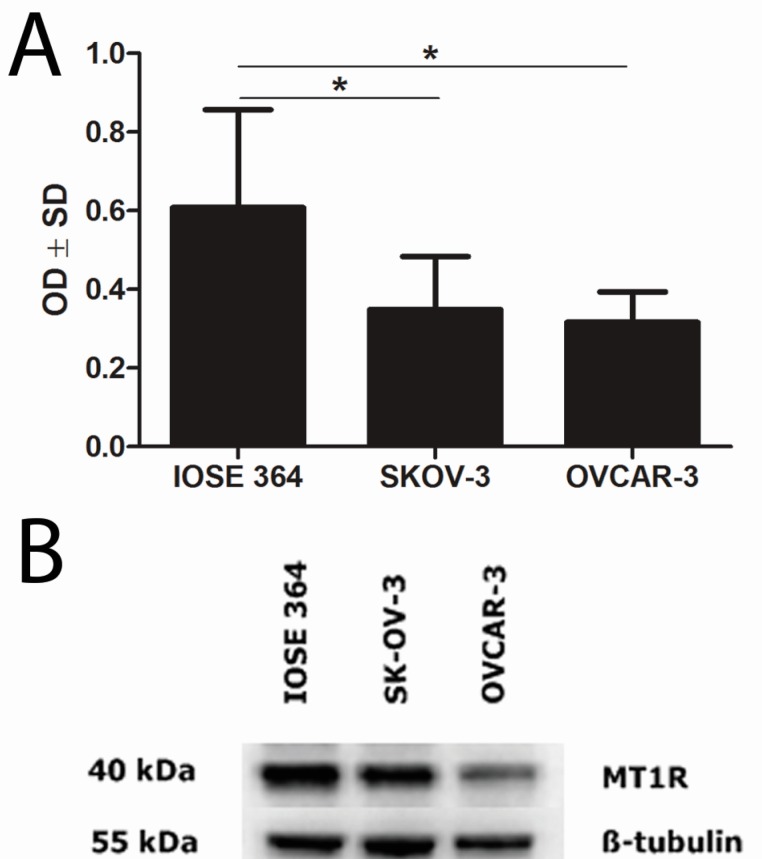
Western blot analysis of MT1 expression in ovarian cell lines (**B**). Densitometric measurement of the bands demonstrates higher MT1 protein contents in normal ovarian cell line (IOSE 364) than in ovarian cancer cell lines (SKOV-3 and OVCAR-3) (**A**). Statistically significant (* *p* < 0.05) differences were noted between cell lines IOSE 364 and SKOV-3, IOSE 364 and OVCAR-3. OD: optical density; SD: standard deviation.

Immunofluorescence demonstrated MT1_CM_ expression in ovarian cell lines (Figure 2). Densitometric analysis of western blot bands illustrated differences in MT1 protein contents between the IOSE 364 (OD (optical density) = 63), SK-OV-3 (OD = 33), and OVCAR-3 (OD = 32) cell lines. Significant differences in MT1 protein level were noted between the cell lines: IOSE 364 and SKOV-3, IOSE 364 and OVCAR-3. The results are presented as a means of percentage values of MT1/β-tubulin OD ratios from three independent measurements (Figure 3).

### 2.2. Discussion

Abundant lines of evidence suggest that Mel exhibits anticancer properties. The earliest observations on the impact of pineal extracts and pinealectomy on tumor growth and metastasis of cancer cells occurred even prior to the discovery of Mel [37,38]. Several *in vitro* and *in vivo* studies point to the antiproliferative activity of Mel in human breast cancer cells, human prostate cancer cells, and melanoma [17,39,40,41]. However, little research has been devoted to the importance of Mel in OC cells or to the importance of Mel receptors in the mechanism of its action. The results of *in vitro* studies on OC cell lines are highly ambiguous. At concentrations of 10^−9^–10^−7^, Mel has been observed to cause 20%–25% growth reduction in BG-1 (ovarian adenocarcinoma cell line) [12]. Tests on seven primary OC cell lines reveled divergent effects: the growth of one cell line was inhibited by 30% at concentrations of 10^−10^–10^−6^ M of Mel, while the other cell line was inhibited by 90% at a concentration of 10^−8^ M of Mel [34]. Mel has no antiproliferative effect on the OVACAR-3 and HTOA OC cell lines alone [35]. On the other hand, this hormone increases the sensitivity of these cell lines to diamminedichloroplatinum (CDDP) chemotherapeutic [35]. Petranka *et al.* have been the only ones to attempt to explain the mechanism of the oncostatic action of Mel in OC cells [12], showing that the anticancer action of Mel in BG1 cells does not result from the interaction of this hormone with either Mel receptor MT2 or with the nuclear receptors RZR/ROR [12]. Mel action as a free radical scavenger, or interacting with calmodulin, was suggested [12]. Yet to date the role of MT1 in the OC has not been studied.

In 1978, Cohen *et al.* were the first to demonstrate the binding of [^3^H]-Mel in the ovaries of hamsters, rats, and humans [42]. This was subsequently confirmed by experiments with 2-[^125^I]-iodomelatonin [29,43]. In our study, MT1 expression was noted in all the studied tumors, as well as in all of the analyzed cell lines (SK-OV-3, OVCAR-3 and IOSE 364). Due the ambiguous nature of the immunohistochemistry (IHC) images, we decided to evaluate the reactions as the cytoplasmic-membrane MT1_CM_ and membrane MT1_M_ fractions of expression. We found a strong correlation between MT1_CM_ and MT1_M_ in all studied cases, which demonstrates the high similarity of both IHC evaluation methods for MT1. Images (IHC, immunofluorscence (IF)) showing MT1_CM_ localization are similar to those obtained in previous publications on breast cancer cells [8,44]. The IHC evaluation shows high MT1 expression in most study cases of OC. This is in line with other studies that show increased MT1 expression in the majority of the examined breast cancer cases [8,44,45,46,47]. So far, an inverse correlation of MT1 expression with malignancy grade G was reported in breast cancer cells on the mRNA and protein levels [8]. In our study, however, no correlation was shown between MT1_CM_ or MT1_M_ and malignancy grade in the WG and SG. There is only one publication, based on the analysis of seven OC cases with clinical data, which disclosed no correlation between sensitivity towards Mel and hormonal receptor status (ER: estrogen receptor; PR: progesterone receptor), patient age, clinical advancement stage, and malignancy grade [34]. Similarly, our study revealed no significant relationships between MT1_CM_ and MT1_M_expression and tumor type, stage of clinical advancement, and presence of residual disease following surgical tumor debulking. In previous studies performed on breast cancer, we also failed to find any significant associations between MT1 expression and age, menopausal status, tumor size, or PR status [8].

The proliferation index of cancer cells based on the evaluation of Ki-67 expression is considered as an indicator of aggressiveness in ovarian tumors and an additional useful prognostic marker [48]. We observed the significantly higher expression of Ki-67 in G3 *vs.* G1 in WG. From our study, it is also apparent that patients without lymph node metastasis (pN-) were characterized by decreased expression of MT1_M_ and Ki-67. It may be assumed that the low risk of lymph node metastases in OC is related not only to the low expression of Ki-67 [49] but also to MT1.

Endogenous Mel levels decrease with age, but it is still unknown whether this is associated with a reduction in MT1 expression. In recent studies, MT1 and Mel levels decreased during aging and during the progression of Alzheimer’s disease [50,51]. Marina Sánchez-Hidalgo *et al.* found, for the first time, reduced *MT1* mRNA expression in the spleen, kidney, liver, and heart in middle-aged rats, compared with young control rats [52]. Only in the thymus were *MT1* mRNA expression levels significantly higher in elderly rats [53]. We also found that immunoexpression of MT1_CM_ and MT1_M_ was significantly higher in patients aged more than 50 years. In our opinion, this change may be the effect of the functional connections between Mel and endocrine ovarian activity.

Previous studies revealed that Mel administration significantly increased the survival time of animals with untreated breast cancer, compared to the control group [54]. Initial clinical studies suggest that Mel reduces the risk of death of patients with cancer in the first year with or without chemotherapy [55]. In one of our studies, we demonstrated that higher MT1 expression is significantly associated with longer OS and event-free survival (EFS) in patients with ER+ breast cancer [8]. However, in our study, the expression level of MT1_CM_ and MT1_M_ was not significantly associated with patient survival time. Analysis of the expression of other markers and patients’ clinical and pathological characteristics in regard to their impact on patient outcome revealed that advanced clinical stage, high Ki-67 antigen expression, and nonoptimal cytoreduction were associated with poor OS in WG and SG. In many reports, high levels of expression of Ki-67 were associated with a shorter survival or shorter disease-free survival [56]. The size of the residual tumor is one of the most important prognostic markers in OC. It was confirmed that the removal of most or all of the tumor lesion (cytoreduction) significantly affects the survival of patients with advanced OC [57].

SK-OV-3 and OVCAR-3 were the first ovarian cell lines in which MT1 was identified [58]. Using the western blot technique, we showed higher MT1 level in the normal cell line IOSE 364 than in both cancer cell lines. We confirmed the results of previous reports showing higher expression of MT1 in the OC cell line SK-OV-3 (ER−), as compared to the OVCAR-3 (ER+) [58]. Treeck *et al.* indicated the potential interaction between Mel and estrogen-signaling pathways. Mel, through MT1, may reduce the expression of ER, inhibit the binding of the ER-estradiol complex to Estrogen Response Element (ERE) on DNA, and affect estrogen-controlled proteins [39,40,59,60]. Treatment with antiestrogens increased MT1 expression in the OVCAR-3 (ER+) cell line and had an effect on ER cell lines SK-OV-3 [58]. Lai *et al.* showed an inverse correlation between MT1 expression and ER receptors in human breast cancer, while Dillon *et al.* found no associations of these antigens [44,46]. Interestingly, in our previous work, we received contrary results with higher MT1 expression in ER+ breast cancer cells [8]. Furthermore, multivariate analysis showed that MT1 was an independent prognostic factor for OS and EFS in the ER+ tumors [8]. The evaluation of the prognostic and predictive role of MT1 in ovarian cancer indicates its limited contribution to this type of tumor.

## 3. Experimental Section

### 3.1. Patients and Tissue Samples

The 84 archival paraffin-embedded OC cases, with their clinical and pathological data, were obtained from the Department of Gynecological Oncology of the University of Medical Sciences in Poznań. The histological types and malignancy grade (G) of the tumors were determined according to the World Health Organization (WHO) criteria in the routinely 6-µm-thick hematoxylin and eosin (H&E) stained sections. Four histological types were diagnosed: serous (65 cases), endometrioid (14 cases), mucinous (3 cases), and clear-cell (2 cases). All the clinical and pathological data are summarized in Table 2. All study material was obtained from patients aged 31 to 79 years (mean age 50). At the time of diagnosis, 60 out of 84 were classified as advanced cases (in stage III or IV of clinical advancement). In the overwhelming number of patients, the debulking surgery (cytoreduction) was not optimal, and presence of residual disease was confirmed histopathologically (63 out of 84). Almost all patients (81 out of 84) showed lymph nodes free of metastatic lesions (pN−). Patients were followed for up for 41.5 (range 4–98) months. During this time, 65 patients (77.38%) died of the disease. The study protocol was approved by the Bioethical Committee of the Wrocław Medical University.

### 3.2. Cell Lines

Molecular studies were performed using two human OC cell lines: SK-OV-3 (European Collection of Cell Culture, Salisbury, UK), OVCAR-3 (American Type Culture Collection, Manassas, VA, USA), and normal ovarian cell line: IOSE 364 (a kind gift from Nelly Auersperg of the Canadian Ovarian Tissue Bank). Both SK-OV-3 and OVCAR-3 manifest epithelial phenotype, but SK-OV-3 has more invasive potential. The IOSE 364 cell line was cultured in medium MCDB 105 and 199 in the ratio 1:1 (Sigma, St. Louis, MO, USA), supplemented with 5% fetal bovine serum (Lonza, Allendale, NJ, USA). SK-OV-3 was grown in McCoy’s 5A medium (Sigma) supplemented with 15% fetal bovine serum (Lonza) and 0.01 mg/mL insulin (Sigma). OCVAR-3 line was cultured in Dulbecco’s Modified Eagle medium (Sigma), supplemented with 15% fetal bovine serum (Lonza). Media were enriched with 1% l-glutamine, penicillin, and streptomycin (Sigma). All cells were cultured in 5% CO_2_ at 37 °C.

**Table 2:** Clinical and pathological characteristics of 84 studied patients.

Parameters	All Patients		Patients with Serous Cancers	
	*N* = 84	Percent (%)	*N* = 65	Percent (%)
Tumor Type				
serous	65	77.3	65	100
endometrioid	14	16.7	−	−
clear-cell	2	2.4	−	−
mucinous	3	3.6	−	−
Age				
≤50	37	44	27	41.5
>50	47	56	38	58.5
Stage				
I, II	20	23.8	11	16.9
III, IV	64	76.2	54	83.1
Tumor Grade				
G1	13	15.5	7	10.8
G2	35	41.6	29	44.6
G3	36	42.9	29	44.6
Lymph Nodes				
pN+	3	3.6	0	0
pN−	81	96.4	65	100
Cytoreduction				
optimal	21	25	13	20
nonoptimal	63	75	52	80
Ki-67				
≤50%	42	50	35	53.8
>50%	42	50	30	46.2

### 3.3. Immunohistochemistry (IHC)

The collected OC specimens were fixed in 4% formalin and embedded in paraffin blocks. The IHC reactions were performed in the Autostainer Link 48 (Dako, Glostrup, Denmark) using an EnVision FLEX visualization system and High pH (Dako). The sections were deparaffinized and their epitopes were exposed using PT-link instrument, in Target Retrieval Solution, High pH buffer (Dako) at 97 °C for 20 min. The expression of MT1 was determined by incubation with noncommercial primary anti-MT1 antibody (rabbit polyclonal antibody, diluted 1:3200; Invitrogen, Carlsbad, CA, USA) for 20 min at room temperature (RT). In order to obtain the serum containing the primary anti-MT1 antibody, the animals were immunized with peptide 536 [61]. The estimation of Ki-67 antigen expression was based on the standard IHC procedure of the EnVision kit, FLEX, Low pH (Dako), as recommended by the manufacturer. Ki-67 was localized using mouse monoclonal primary antibody (clone MIB-1; Dako), diluted 1:100 and incubated for 20 min at RT. Secondary goat antirabbit immunoglobulin antibodies (EnVision/HRP; Dako) were linked to the horseradish peroxidase. The substrate for the reaction was DAB (3,3'-diaminobenzidine tetrachlorohydrate). All slides were counterstained with hematoxylin (Dako). In the negative control preparations primary antibodies were omitted.

### 3.4. Evaluation of IHC Reaction

The evaluation of the IHC reaction was conducted by two independent investigators (PD, BP) using a BX-41 light microscope (Olympus, Tokyo, Japan). The evaluation of cytoplasmatic/membranous MT1_CM_ expression was performed using the semiquantitative Immunoreactive Score (IRS) of Remmele and Stegner [62], with our own modifications. The scale takes into account the percentage of cells with a noticeable color reaction (A) and the intensity of this reaction (B). The final score, calculated as the product of the two values (A × B), ranges between 0 and 15 (Table 3). The assessment of MT1_M_ was performed using the IRS scale of 0–5 points for the percentage of positive cells. For MT1_CM_, samples receiving 0–5 points were considered to display weak expression, while those receiving 6–15 points showed strong expression. For MT1_M_, the ranges 0–1 and 2–5, respectively, were employed. The intensity of Ki-67 antigen expression in tumor cells was evaluated according to the percentage of positive tumor cells, as compared to all tumor cells: 0 points: no reaction; 1 point: 1%–10%; 2 points: 11%–25%; 3 points: 26%–50%; 4 points: >50% [63]. The Ki-67 index was taken to indicate low expression when the percentage of positive cells was ≤50%, and high when this was >50%.

**Table 3:** Semiquantitative Immunoreactive Score (IRS) of Remmele and Stegner [57] in our own modification [8]. Expression level of MT1_CM_ was evaluated on a scale from 0 to 15 points (A × B).

Points	A	Points	B
	Percentage of Positive Cells		Intensity of Color Reaction
0	No positive cell	0	No color reaction
1	<10%	1	Low
2	11%–25%	2	Moderate
3	26%–50%	3	High
4	51%–80%		
5	>80%		

### 3.5. Western Blot

IOSE 364, SK-OV-3, and OVCAR-3 cell lines were subjected to trypsinization and scored in a hemocytometer. For each test, 5 × 10^6^–10 × 10^6^ tumor cells were sampled in the exponential growth phase. After washing in cold PBS (phosphate buffered saline), the cells were subjected to lysis for 20 min in ice with the addition of RIPA buffer (50 mM Tris-Cl pH 8.0, 150 mM NaCl, 0.1% SDS, 1% Igepal CA-630; Sigma), 0.5% sodium deoxycholate (Sigma), a cocktail of protease inhibitors (Sigma), and 0.5 mM PMSF (phenylmethanesulfonylfluoride) (Sigma). Protein concentration was measured using the BCA (bicinchoninic acid assay) technique (Thermo-Pierce, Rockford, IL, USA) and a NanoDrop 1000 spectrophotometer (Thermo Scientific, Waltham, MA, USA). Cell extracts were mixed with a sample buffer (250 mM TRIS pH 6.8, 40% glycerol, 20% (*v*/*v*) β-mercaptoethanol, 100 mM DTT (dithiothreitol), 0.33 mg/mL bromophenol blue, 8% SDS (sodium dodecyl sulfate)) and subjected to denaturation for 10 min at 95 °C. Equal amounts of protein (40 µg per lane) were separated by electrophoresis following Laemmli [64] in a 10% gel using a Mini Protean 3 apparatus (Bio-Rad, Marnes-la-Coquette, France). Subsequently, the proteins were electrophoretically transferred to PVDF (polyvinylidene difluoride) membrane (Immobilon P; Millipore, Billerica, MA, USA) and the sites of nonspecific binding were blocked using 5% skimmed milk (Bio-Rad) in TBS (tris-buffered saline) buffer. The expression of MT1 was detected using polyclonal anti-MT1 antibody (Invitrogen). Incubation was conducted for 16 h at 4 °C with delicate shaking, in the solution of the antibody diluted 1:3500 in 0.5% milk, in 0.2% TBST (tris-buffered saline and 20% tween). The membrane was fourfold rinsed in 0.2% TBST buffer and subsequently incubated for 1 h in a solution of donkey antirabbit antibody, conjugated with peroxidase (1:2000; Jackson Immunoresearch, Suffolk, UK). Detection was performed using a substrate for chemiluminescence (Immun-Star HRP Chemiluminescent Kit; Bio-Rad), and the results were documented for exposure times ranging from 2 s to 30 min in a Chemi-Doc XRS Molecular Imager apparatus (Bio-Rad). The resulting bands were estimated by densitometric quantitative analysis of proteins and normalized to β-tubulin (Abcam, Cambridge, UK) levels. The amount of the applied protein was controlled by staining the total protein on the membrane using Ponceau S (Sigma).

### 3.6. Immunofluorscence (IF)

Cells were fixed in 4% formaldehyde at RT. The membranes were permeabilized using 0.2% Triton X-100 (Sigma). Cells were incubated with primary antibody anti-MT1 diluted 1:3200 (Invitrogen). Antirabbit secondary FITC-conjugated antibody (Invitrogen) at a concentration of 1:2000 was then added for 20 min at RT. After rinsing, the cells were mounted in DAPI-containing medium (Invitrogen). The preparations were analyzed using a BX51 fluorescence microscope and CellF software (Olympus).

### 3.7. Statistical Analysis

The results were subjected to statistical analysis using Prism 5.0 software (GraphPad, La Jolla, CA, USA). The expression of MT1 was analyzed in regard to the clinical and pathological data utilizing the Kruskal–Wallis and Mann–Whitney tests. Correlation analysis was performed using Spearman’s rank correlation test. The Kaplan–Meier method and the log-rank test were used to determine the impact of particular characteristics on the patients’ overall survival (OS). Multivariate analysis was performed using the Cox regression model. Bonferroni’s Multiple Comparison Test was used to analyze the difference in the MT1 protein contents between cell lines. For each variable, the hazard ratio and the 95% confidence interval (CI) were determined. The results were considered statistically significant for *p* < 0.05.

## 4. Conclusions

Numerous reports suggest the involvement of Mel in ovarian physiology (in ovulation, follicular development, luteal function), as well as in the pathophysiology of endometriosis, polycystic ovaries syndrome, and cancer [30]. The evidence that Mel acts through MT1 in the case of OC is equivocal. The present study provides evidence that MT1 is expressed in both OC cells of clinical specimens and in the ovarian cell lines IOSE 364, SK-OV-3, OVCAR-3. We demonstrated for the first time the expression of MT1 in a large number of OC cases, and correlated the obtained results with patients’ clinical and pathological data. We have shown that both evaluation methods of IHC reactions (cytoplasmic-membrane MT1_CM_ and membrane MT1_M_) strongly correlate. MT1 expression was higher in patients over fifty years old and correlated with Ki-67 antigen expression. Higher expression of MT1 was noted in the ER-negative SK-OV-3 cell line than in the ER-positive OVCAR-3. No significant relationship was found between MT1_CM_ and MT1_M_ expression with tumor malignancy grade, clinical advancement stage, tumor type, presence of residual disease following cytoreduction, or patient survival. Our results suggest the limited prognostic significance of MT1 expression in OC cells. The antiproliferative effect of Mel on OC cells may be the result of a receptor-independent mechanism.
